# 4-Hydroxy-2,2,6,6-tetra­methyl­piperidinium trifluoro­acetate

**DOI:** 10.1107/S1600536807066305

**Published:** 2007-12-18

**Authors:** Yan-Xue Chen, Mei-Ling Han, Yi Deng, Jin-Hui Yang

**Affiliations:** a School of Chemical Engineering and Technology, Tianjin University, Tianjin 300072, People’s Republic of China; b School of Pharmaceutical Science and Technology, Tianjin University, Tianjin 300072, People’s Republic of China; c School of Materials Science and Engineering, Shijizhuang Railway Institute, Shijiazhuang 050043, People’s Republic of China

## Abstract

The title compound, C_9_H_20_NO^+^·C_2_F_3_O_2_^−^, is an important inter­mediate in the synthesis of hindered light stabilizers. The piperidinium ring adopts a chair conformation with the hydroxyl group in an equatorial position. The crystal packing is stabilized by O—H⋯O and N—H⋯O hydrogen bonds. The CF_3_ group is disordered over two positions with almost equal site occupancy factors.

## Related literature

For general background, see: Borzatta & Carrozza (1991 ▶). For related structures, see: Nengfang *et al.* (2005 ▶).
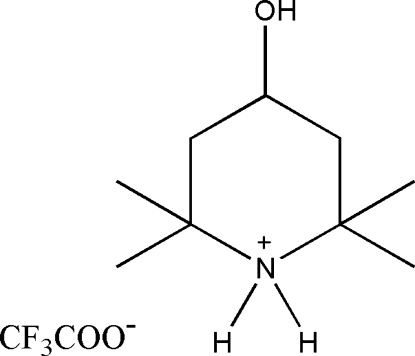


## Experimental

### 

#### Crystal data

C_9_H_20_NO^+^·C_2_F_3_O_2_^−^
*M* *_r_* = 271.28
Orthorhombic, 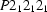

*a* = 7.6204 (8) Å
*b* = 9.8939 (10) Å
*c* = 18.099 (2) Å
*V* = 1364.6 (2) Å^3^
*Z* = 4
Mo *K*α radiation
μ = 0.12 mm^−1^
*T* = 113 (2) K
0.22 × 0.20 × 0.16 mm

#### Data collection

Rigaku Saturn diffractometer
Absorption correction: multi-scan (*CrystalClear*; Rigaku/MSC, 2005 ▶) *T*_min_ = 0.974, *T*_max_ = 0.981
17989 measured reflections
2039 independent reflections
1993 reflections with *I* > 2σ(*I*)
*R*_int_ = 0.057

#### Refinement

*R*[*F* ^2^ > 2σ(*F* ^2^)] = 0.057
*wR*(*F* ^2^) = 0.132
*S* = 1.23
2039 reflections
202 parameters
48 restraints
H-atom parameters constrained
Δρ_max_ = 0.22 e Å^−3^
Δρ_min_ = −0.21 e Å^−3^

### 

Data collection: *CrystalClear* (Rigaku/MSC, 2005 ▶); cell refinement: *CrystalClear*; data reduction: *CrystalClear*; program(s) used to solve structure: *SHELXS97* (Sheldrick, 1997 ▶); program(s) used to refine structure: *SHELXL97* (Sheldrick, 1997 ▶); molecular graphics: *SHELXTL* (Bruker, 1997 ▶); software used to prepare material for publication: *CrystalStructure* (Rigaku/MSC, 2005 ▶).

## Supplementary Material

Crystal structure: contains datablocks I, global. DOI: 10.1107/S1600536807066305/bt2664sup1.cif

Structure factors: contains datablocks I. DOI: 10.1107/S1600536807066305/bt2664Isup2.hkl

Additional supplementary materials:  crystallographic information; 3D view; checkCIF report

## Figures and Tables

**Table 1:** Hydrogen-bond geometry (Å, °)

*D*—H⋯*A*	*D*—H	H⋯*A*	*D*⋯*A*	*D*—H⋯*A*
N1—H1*B*⋯O2	0.92	1.88	2.786 (3)	169
N1—H1*A*⋯O1^i^	0.92	1.96	2.869 (3)	171
O1—H1⋯O3^ii^	0.84	1.85	2.682 (3)	171

Symmetry codes: (i) 
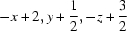
; (ii) 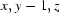.

**Fig. 1.**
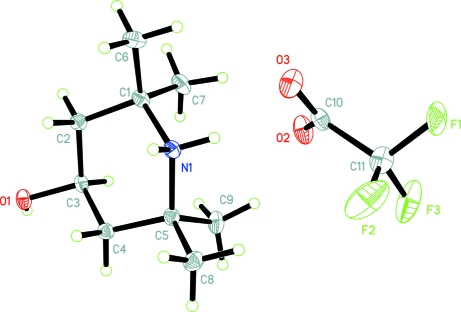
A perspective view of the title compound. Displacement ellipsoids are drawn at the 30% probability level and H atoms are shown as small spheres of arbitrary radii. Only the major occupied site of the disordered CF3 group is shown.

## supplementary crystallographic information

### Comment

4-hydroxyl-2,2,6,6-tetramethylpiperidine is a very important intermediate in the synthesis of hindered light stabilizers (Borzatta & Carrozza, 1991; She *et al.*, 2005). The piperidium ring adopts a chair conformation with the hydroxyl group in an equatorial position. The crystal packing is stabilized by O—H···O and N—H···O hydrogen bonds.

### Experimental

An ethanol solution (10 ml) of 2,2,6,6-tetramethylpiperidin-4-ol (3.2 mmol, 0.5 g) was added dropwise to a stirred aqueous solution (6 ml) of trifluoroacetic acid (3.8 mmol, 0.43 g) at 293 K. Then the reaction mixture was filtered and the filtrate stood for about five days until colourless needle shaped crystals were obtained.

### Refinement

In the absence of anomalous scatterers, Friedel pairs had been merged and the absolute structure was arbitrarily assigned. All H atoms were positioned geometrically with C—H ranging from 0.98Å to 1.00Å and refined as riding with *U*_iso_(H)=1.2*U*_eq_(C,*N*,*O*) or 1.5_eq_(C_methyl_). The CF_3_ group is disordered over two position with a ratio of occupancy factors of 0.459 (1)/0.541 (1). The atoms of the CF_3_ group were restrained to an isotropic behaviour.

### Crystal data

**Table:**

C_9_H_20_NO^+^·C_2_F_3_O_2_^–^	*D*_x_ = 1.320 Mg m^−^^3^
*M**_r_* = 271.28	Mo *K*α radiation λ = 0.71070 Å
Orthorhombic, *P*2_1_2_1_2_1_	Cell parameters from 4344 reflections
*a* = 7.6204 (8) Å	θ = 2.1–28.7º
*b* = 9.8939 (10) Å	µ = 0.12 mm^−^^1^
*c* = 18.099 (2) Å	*T* = 113 (2) K
*V* = 1364.6 (2) Å^3^	Needle, colourless
*Z* = 4	0.22 × 0.20 × 0.16 mm
*F*_000_ = 576	

### Data collection

**Table:**

Rigaku Saturn diffractometer	2039 independent reflections
Radiation source: rotating anode	1993 reflections with *I* > 2σ(*I*)
Monochromator: confocal	*R*_int_ = 0.057
Detector resolution: 14.63 pixels mm^-1^	θ_max_ = 28.7º
*T* = 113(2) K	θ_min_ = 2.3º
ω scans	*h* = −10→10
Absorption correction: multi-scan(CrystalClear; Rigaku/MSC, 2005)	*k* = −13→13
*T*_min_ = 0.974, *T*_max_ = 0.981	*l* = −24→24
17989 measured reflections	

### Refinement

**Table:**

Refinement on *F*^2^	Secondary atom site location: difference Fourier map
Least-squares matrix: full	Hydrogen site location: inferred from neighbouring sites
*R*[*F*^2^ > 2σ(*F*^2^)] = 0.057	H-atom parameters constrained
*wR*(*F*^2^) = 0.132	*w* = 1/[σ^2^(*F*_o_^2^) + (0.0506*P*)^2^ + 0.4132*P*] where *P* = (*F*_o_^2^ + 2*F*_c_^2^)/3
*S* = 1.23	(Δ/σ)_max_ = 0.001
2039 reflections	Δρ_max_ = 0.22 e Å^−^^3^
202 parameters	Δρ_min_ = −0.21 e Å^−^^3^
48 restraints	Extinction correction: SHELXL, Fc^*^=kFc[1+0.001xFc^2^λ^3^/sin(2θ)]^-1/4^
Primary atom site location: structure-invariant direct methods	Extinction coefficient: 0.028 (4)

### Special details

**Table:**

Geometry. All e.s.d.'s (except the e.s.d. in the dihedral angle between two l.s. planes) are estimated using the full covariance matrix. The cell e.s.d.'s are taken into account individually in the estimation of e.s.d.'s in distances, angles and torsion angles; correlations between e.s.d.'s in cell parameters are only used when they are defined by crystal symmetry. An approximate (isotropic) treatment of cell e.s.d.'s is used for estimating e.s.d.'s involving l.s. planes.
Refinement. Refinement of *F*^2^ against ALL reflections. The weighted *R*-factor *wR* and goodness of fit *S* are based on *F*^2^, conventional *R*-factors *R* are based on *F*, with *F* set to zero for negative *F*^2^. The threshold expression of *F*^2^ > σ(*F*^2^) is used only for calculating *R*-factors(gt) *etc*. and is not relevant to the choice of reflections for refinement. *R*-factors based on *F*^2^ are statistically about twice as large as those based on *F*, and *R*- factors based on ALL data will be even larger.

### Fractional atomic coordinates and isotropic or equivalent isotropic displacement parameters (Å^2^)

**Table:**

	*x*	*y*	*z*	*U*_iso_*/*U*_eq_	Occ. (<1)
O1	0.9028 (3)	−0.20835 (18)	0.73102 (11)	0.0271 (5)	
H1	0.8683	−0.2769	0.7078	0.041*	
O2	0.6874 (3)	0.4099 (2)	0.58896 (13)	0.0394 (6)	
O3	0.7885 (3)	0.5590 (2)	0.67201 (12)	0.0398 (6)	
N1	0.8175 (3)	0.1997 (2)	0.67551 (12)	0.0226 (5)	
H1A	0.9012	0.2378	0.7056	0.027*	
H1B	0.7631	0.2695	0.6510	0.027*	
C1	0.6801 (4)	0.1339 (3)	0.72563 (15)	0.0244 (5)	
C2	0.7571 (4)	0.0022 (3)	0.75609 (14)	0.0241 (5)	
H2A	0.8503	0.0245	0.7922	0.029*	
H2B	0.6639	−0.0475	0.7827	0.029*	
C3	0.8336 (4)	−0.0893 (3)	0.69685 (14)	0.0233 (5)	
H3	0.7392	−0.1152	0.6612	0.028*	
C4	0.9789 (4)	−0.0143 (3)	0.65571 (15)	0.0234 (5)	
H4A	1.0300	−0.0753	0.6181	0.028*	
H4B	1.0729	0.0095	0.6911	0.028*	
C5	0.9142 (4)	0.1151 (3)	0.61754 (14)	0.0241 (6)	
C6	0.6475 (4)	0.2352 (3)	0.78794 (18)	0.0326 (7)	
H6A	0.7557	0.2477	0.8164	0.049*	
H6B	0.5549	0.2009	0.8205	0.049*	
H6C	0.6110	0.3220	0.7668	0.049*	
C7	0.5089 (4)	0.1126 (3)	0.68236 (19)	0.0335 (7)	
H7A	0.4141	0.0899	0.7168	0.050*	
H7B	0.5244	0.0387	0.6469	0.050*	
H7C	0.4789	0.1958	0.6558	0.050*	
C8	1.0678 (4)	0.2025 (3)	0.59214 (16)	0.0302 (6)	
H8A	1.0229	0.2852	0.5691	0.045*	
H8B	1.1384	0.1522	0.5562	0.045*	
H8C	1.1408	0.2262	0.6348	0.045*	
C9	0.7963 (4)	0.0851 (3)	0.55106 (16)	0.0331 (7)	
H9A	0.7112	0.0149	0.5643	0.050*	
H9B	0.8685	0.0536	0.5097	0.050*	
H9C	0.7338	0.1675	0.5366	0.050*	
C10	0.7562 (4)	0.5187 (3)	0.60881 (16)	0.0281 (6)	
C11	0.8356 (12)	0.6096 (9)	0.5496 (5)	0.037 (3)	0.459 (14)
F1	0.7559 (17)	0.7299 (6)	0.5473 (4)	0.067 (3)	0.459 (14)
F2	1.0045 (10)	0.6341 (15)	0.5611 (4)	0.086 (4)	0.459 (14)
F3	0.8194 (18)	0.5655 (13)	0.4799 (6)	0.070 (4)	0.459 (14)
C11'	0.7828 (13)	0.6165 (8)	0.5453 (4)	0.042 (3)	0.541 (14)
F1'	0.6352 (15)	0.6809 (12)	0.5291 (5)	0.126 (4)	0.541 (14)
F2'	0.900 (2)	0.7116 (8)	0.5599 (3)	0.099 (5)	0.541 (14)
F3'	0.8406 (14)	0.5571 (12)	0.4839 (5)	0.070 (4)	0.541 (14)

### Atomic displacement parameters (Å^2^)

**Table:**

	*U*^11^	*U*^22^	*U*^33^	*U*^12^	*U*^13^	*U*^23^
O1	0.0347 (11)	0.0164 (8)	0.0303 (10)	0.0008 (8)	−0.0069 (9)	0.0017 (8)
O2	0.0504 (13)	0.0219 (10)	0.0458 (12)	−0.0013 (10)	−0.0187 (11)	−0.0011 (9)
O3	0.0615 (15)	0.0274 (10)	0.0306 (11)	−0.0091 (11)	−0.0059 (11)	−0.0024 (9)
N1	0.0236 (11)	0.0177 (10)	0.0266 (10)	−0.0003 (9)	−0.0010 (9)	−0.0003 (9)
C1	0.0212 (12)	0.0193 (11)	0.0327 (14)	−0.0006 (10)	0.0012 (11)	0.0002 (10)
C2	0.0220 (12)	0.0239 (12)	0.0266 (12)	−0.0010 (11)	0.0014 (10)	0.0015 (10)
C3	0.0260 (13)	0.0189 (11)	0.0251 (12)	0.0016 (10)	−0.0050 (10)	0.0015 (10)
C4	0.0242 (12)	0.0226 (12)	0.0235 (12)	0.0017 (11)	−0.0001 (10)	−0.0021 (10)
C5	0.0278 (13)	0.0202 (12)	0.0244 (12)	0.0020 (11)	−0.0004 (11)	−0.0008 (10)
C6	0.0298 (15)	0.0269 (14)	0.0412 (16)	0.0004 (12)	0.0098 (13)	−0.0047 (13)
C7	0.0242 (13)	0.0266 (13)	0.0498 (18)	0.0009 (11)	−0.0036 (13)	0.0032 (14)
C8	0.0356 (16)	0.0263 (14)	0.0286 (14)	−0.0029 (13)	0.0046 (12)	0.0016 (12)
C9	0.0434 (18)	0.0272 (14)	0.0287 (14)	0.0013 (13)	−0.0109 (13)	0.0016 (11)
C10	0.0292 (13)	0.0200 (12)	0.0351 (14)	0.0032 (11)	−0.0061 (12)	−0.0015 (11)
C11	0.048 (5)	0.030 (5)	0.033 (5)	−0.009 (4)	−0.006 (4)	−0.004 (4)
F1	0.132 (8)	0.020 (3)	0.049 (4)	0.017 (4)	0.014 (4)	0.011 (2)
F2	0.064 (5)	0.129 (9)	0.065 (4)	−0.058 (5)	0.007 (3)	0.005 (5)
F3	0.136 (10)	0.043 (6)	0.030 (5)	−0.037 (6)	−0.025 (5)	0.002 (4)
C11'	0.063 (6)	0.037 (4)	0.026 (4)	0.000 (4)	−0.010 (3)	−0.001 (3)
F1'	0.123 (7)	0.136 (7)	0.120 (6)	0.051 (6)	−0.004 (5)	0.080 (5)
F2'	0.198 (13)	0.060 (5)	0.039 (3)	−0.082 (7)	0.013 (6)	−0.012 (3)
F3'	0.105 (7)	0.063 (7)	0.042 (5)	−0.025 (5)	0.042 (4)	−0.024 (4)

### Geometric parameters (Å, °)

**Table:**

O1—C3	1.431 (3)	C6—H6A	0.9800
O1—H1	0.8400	C6—H6B	0.9800
O2—C10	1.250 (3)	C6—H6C	0.9800
O3—C10	1.236 (3)	C7—H7A	0.9800
N1—C1	1.531 (3)	C7—H7B	0.9800
N1—C5	1.531 (3)	C7—H7C	0.9800
N1—H1A	0.9200	C8—H8A	0.9800
N1—H1B	0.9200	C8—H8B	0.9800
C1—C6	1.529 (4)	C8—H8C	0.9800
C1—C2	1.531 (4)	C9—H9A	0.9800
C1—C7	1.537 (4)	C9—H9B	0.9800
C2—C3	1.519 (4)	C9—H9C	0.9800
C2—H2A	0.9900	C10—C11'	1.516 (7)
C2—H2B	0.9900	C10—C11	1.525 (8)
C3—C4	1.526 (4)	C11—F2	1.326 (9)
C3—H3	1.0000	C11—F1	1.337 (8)
C4—C5	1.536 (4)	C11—F3	1.339 (8)
C4—H4A	0.9900	C11'—F2'	1.324 (8)
C4—H4B	0.9900	C11'—F1'	1.325 (8)
C5—C8	1.527 (4)	C11'—F3'	1.333 (8)
C5—C9	1.530 (4)		
			
C3—O1—H1	109.5	H6A—C6—H6B	109.5
C1—N1—C5	120.2 (2)	C1—C6—H6C	109.5
C1—N1—H1A	107.3	H6A—C6—H6C	109.5
C5—N1—H1A	107.3	H6B—C6—H6C	109.5
C1—N1—H1B	107.3	C1—C7—H7A	109.5
C5—N1—H1B	107.3	C1—C7—H7B	109.5
H1A—N1—H1B	106.9	H7A—C7—H7B	109.5
C6—C1—N1	105.6 (2)	C1—C7—H7C	109.5
C6—C1—C2	110.8 (2)	H7A—C7—H7C	109.5
N1—C1—C2	108.2 (2)	H7B—C7—H7C	109.5
C6—C1—C7	109.1 (2)	C5—C8—H8A	109.5
N1—C1—C7	109.7 (2)	C5—C8—H8B	109.5
C2—C1—C7	113.1 (2)	H8A—C8—H8B	109.5
C3—C2—C1	113.6 (2)	C5—C8—H8C	109.5
C3—C2—H2A	108.9	H8A—C8—H8C	109.5
C1—C2—H2A	108.9	H8B—C8—H8C	109.5
C3—C2—H2B	108.9	C5—C9—H9A	109.5
C1—C2—H2B	108.9	C5—C9—H9B	109.5
H2A—C2—H2B	107.7	H9A—C9—H9B	109.5
O1—C3—C2	109.1 (2)	C5—C9—H9C	109.5
O1—C3—C4	110.1 (2)	H9A—C9—H9C	109.5
C2—C3—C4	109.5 (2)	H9B—C9—H9C	109.5
O1—C3—H3	109.4	O3—C10—O2	128.9 (3)
C2—C3—H3	109.4	O3—C10—C11'	117.9 (4)
C4—C3—H3	109.4	O2—C10—C11'	112.8 (4)
C3—C4—C5	113.1 (2)	O3—C10—C11	112.4 (4)
C3—C4—H4A	109.0	O2—C10—C11	118.2 (4)
C5—C4—H4A	109.0	F2—C11—F1	106.4 (8)
C3—C4—H4B	109.0	F2—C11—F3	107.3 (8)
C5—C4—H4B	109.0	F1—C11—F3	102.6 (8)
H4A—C4—H4B	107.8	F2—C11—C10	112.5 (7)
C8—C5—C9	108.9 (2)	F1—C11—C10	111.5 (7)
C8—C5—N1	105.4 (2)	F3—C11—C10	115.7 (9)
C9—C5—N1	111.2 (2)	F2'—C11'—F1'	106.0 (8)
C8—C5—C4	111.2 (2)	F2'—C11'—F3'	104.8 (8)
C9—C5—C4	112.4 (2)	F1'—C11'—F3'	107.9 (8)
N1—C5—C4	107.6 (2)	F2'—C11'—C10	113.2 (6)
C1—C6—H6A	109.5	F1'—C11'—C10	111.2 (6)
C1—C6—H6B	109.5	F3'—C11'—C10	113.3 (8)
			
C5—N1—C1—C6	165.7 (2)	O2—C10—C11—F2	−121.8 (8)
C5—N1—C1—C2	47.0 (3)	C11'—C10—C11—F2	164 (3)
C5—N1—C1—C7	−76.9 (3)	O3—C10—C11—F1	−69.1 (8)
C6—C1—C2—C3	−166.0 (2)	O2—C10—C11—F1	118.7 (8)
N1—C1—C2—C3	−50.6 (3)	C11'—C10—C11—F1	45 (2)
C7—C1—C2—C3	71.2 (3)	O3—C10—C11—F3	174.2 (8)
C1—C2—C3—O1	179.6 (2)	O2—C10—C11—F3	2.0 (11)
C1—C2—C3—C4	59.1 (3)	C11'—C10—C11—F3	−72 (2)
O1—C3—C4—C5	−180.0 (2)	O3—C10—C11'—F2'	25.7 (9)
C2—C3—C4—C5	−60.1 (3)	O2—C10—C11'—F2'	−160.6 (8)
C1—N1—C5—C8	−166.6 (2)	C11—C10—C11'—F2'	−47 (2)
C1—N1—C5—C9	75.6 (3)	O3—C10—C11'—F1'	−93.5 (8)
C1—N1—C5—C4	−47.8 (3)	O2—C10—C11'—F1'	80.2 (9)
C3—C4—C5—C8	167.3 (2)	C11—C10—C11'—F1'	−167 (3)
C3—C4—C5—C9	−70.4 (3)	O3—C10—C11'—F3'	144.8 (8)
C3—C4—C5—N1	52.4 (3)	O2—C10—C11'—F3'	−41.5 (10)
O3—C10—C11—F2	50.4 (9)	C11—C10—C11'—F3'	72 (2)

### Hydrogen-bond geometry (Å, °)

**Table:**

*D*—H···*A*	*D*—H	H···*A*	*D*···*A*	*D*—H···*A*
N1—H1B···O2	0.92	1.88	2.786 (3)	169
N1—H1A···O1^i^	0.92	1.96	2.869 (3)	171
O1—H1···O3^ii^	0.84	1.85	2.682 (3)	171

Symmetry codes: (i) −*x*+2, *y*+1/2, −*z*+3/2; (ii) *x*, *y*−1, *z*.
